# Ginsenoside Rd Attenuates Tau Phosphorylation in Olfactory Bulb, Spinal Cord, and Telencephalon by Regulating Glycogen Synthase Kinase 3*β* and Cyclin-Dependent Kinase 5

**DOI:** 10.1155/2021/4485957

**Published:** 2021-12-26

**Authors:** Ling Li, Tian Li, Xin Tian, Ling Zhao

**Affiliations:** ^1^Department of Geriatrics, Shaanxi Provincial Hospital of Traditional Chinese Medicine, Xi'an 710003, China; ^2^Department of Cardiology, Shaanxi Provincial Hospital of Traditional Chinese Medicine, Xi'an 710003, China; ^3^Department of Neurology, Xinchang Hospital Affiliated to Wenzhou Medical University, 117 Gushan Middle Rd, Xinchang 312500, China

## Abstract

**Objective:**

Ginseng is a plant of the family Acanthopanaceae. It has been used for thousands of years in China. It is known as the king of hundred herbs. It was recorded first in Shennong Baicao Jing. It has been found that ginsenoside Rd is a neuroprotective agent. This article aims to explore the protective roles of ginsenoside Rd in Alzheimer's disease. Rd, a Chinese herb, may be a promising treatment drug for Alzheimer′s disease (AD) and is also reported to be related to several pathological changes, including the deposition of A*β* and tau hyperphosphorylation in AD as it decreases the deposition of tau hyperphosphorylation in APP transgenic mice.

**Methods:**

In this study, APP transgenic mice were pretreated with 10 mg/kg Rd for six months, and the effect of Rd on neuropathological deficits in the olfactory bulb, spinal cord, and telencephalon of APP transgenic mice was investigated. The phosphorylation levels of tau (S199/202, S396, S404, and Tau5) and the activities of the proteins glycogen synthase kinase 3*β* (Tyr216) and cyclin-dependent kinase 5 (P25/P35) were measured.

**Results:**

The pretreatment of Rd effectively decreased the production and deposition of hyperphosphorylated tau (S199/202, S396, and S404) protein by depressing the expression of glycogen synthase kinase 3*β* (GSK-3*β*/Tyr216) and cyclin-dependent kinase 5 (CDK5/P25).

**Conclusion:**

These findings suggest that ginsenoside Rd could improve the pathological changes of AD in the olfactory bulb, spinal cord, and telencephalon, which further demonstrated the potential therapeutic effect of Rd in early AD.

## 1. Introduction

Alzheimer's disease (AD) is an age-related neurodegenerative disease with clinical manifestations of cognitive impairment and sensory and motor loss. Its pathological manifestations include amyloid beta-senile plaque, nerve fiber tangles, and neuroinflammatory response. Most studies [1] about AD focus on the hippocampus, which is considered to be the main area of cognitive memory. Little attention has been paid to other areas of the central nervous system (CNS), such as the olfactory bulb (OB), spinal cord (SP), and telencephalon (CE). However, studies have shown that beta-amyloid plaques [2], nerve fiber tangles, and hyperphosphorylated tau [3] can accumulate in the spinal cord and telencephalon of AD patients. Similarly, the olfactory system is a unique brain system [4]. Previous studies have shown that olfactory function decreases with age. The decline of olfactory function is a part of the clinical phenotype of neurodegenerative diseases including AD. In AD mice, there was olfactory impairment before the clinical onset of cognitive impairment [5]. Olfactory disorders are common in AD patients because of neuropathological abnormalities in the central and peripheral olfactory systems. Therefore, olfactory dysfunction is considered as an early potential biological marker for the diagnosis of AD [6]. As a cortical sensory structure, the olfactory bulb (OB) is the first synaptic relay station for olfactory perception in the brain, which receives input directly from olfactory neurons in the nasal cavity [7]. Olfaction involves different processes from sensory neurons to the OB, including decoding and plasticity of the prefrontal cortex, and finally the downstream neurons in the hippocampus [8].

Neuropathological studies have found that the underlying causes of AD olfactory dysfunction may be related to the presence of NFT. Aged A*β* plaque exists in the preolfactory nucleus, piriform anterior cortex, endoolfactory cortex, amygdala, and hippocampus [9]. In addition, the presence and severity of beta-amyloid protein and hyperphosphorylated tau in OB can reflect the severity of AD pathology in other brain regions [10]. A*β* and tau pathology of OB can be used as important etiological and diagnostic markers of olfactory dysfunction and AD. Compared with other brain area assessments, detection of AD pathological changes in OB after drug intervention may be a better assessment in the early stage of AD or after short-term treatment. Previous studies using different strains of transgenic mice carrying human amyloid precursor protein and/or presenilin mutations have the mentioned plaque deposition in the spinal cord and telencephalon [11], and entanglement can occur if tau mutations coexist [12]. In addition, the structural advantages of spinal axons may facilitate the detection of previously unreported micropathological amyloid deposition. In fact, we found that amyloid plaques were unevenly distributed along the spinal cord. We also found that the axons of the spinal cord contain fine linear deposition of beta-amyloid protein and changes in the microstructure of the myelin sheath, suggesting that axonal disease and myelin sheath disease, driven by aggregation of beta-amyloid subcells, may play a role in the pathophysiology of AD, not only in the spinal cord, but throughout the central nervous system. However, little is known about the systematic accumulation of olfactory bulb, spinal cord, and telencephalon pathology in AD transgenic mice. The aim of this study was to detect the olfactory bulb, spinal cord, and telencephalon of APP transgenic AD mice in a spatial and temporal manner to evaluate its potential as a pathological development model of AD.

Ginseng (*Panax ginseng Meyer*) is a plant of the family Acanthopanaceae. It has been used for thousands of years in our country. It is known as the king of hundred herbs. It has many effects, such as improving memory, restoring the spirit, stopping palpitation, eliminating evil spirits, enjoying the eyes, and enjoying the wisdom [13]. Recent studies have shown that ginsenosides have multiple protective effects on AD-related animal models *in vitro* and *in vivo* [14, 15]. Ginsenoside has many monomers. Ginsenoside Rd (Dammar-24(25)-ene- 3b,12b,20(S)-triol-(20-O-b-D-glucopyranosyl-(1,2)-b-D-glucopyranoside) is considered to be one of the important components of ginseng and *Panax notoginseng*. The content of Rd is generally low in plants, but Rd is one of the main forms of some major saponins after metabolism, such as intestinal enzymes which can metabolize Rb1 to produce Rd. It has been found that Rd acts as a neuroprotective agent through anti-inflammation, antioxidation, antiapoptosis, and inhibition of calcium influx [16, 17]. Furthermore, ginsenoside Rd significantly reduced the number and size of metastatic nodules of liver, lung, and kidney tumors in mice with metastasis [18]. Also, ginsenoside Rd improved cardiac dysfunction and remodeling caused by pressure overload, which is related to the inhibition of multiple signaling pathways [19].

Our study showed that ginsenoside Rd could improve the learning and memory ability of rats and had neuroprotective effect on acute brain injury induced by A*β*_1-40_ in adult rats [20]. In order to further elucidate the neuroprotective effect of Rd on AD and its possible mechanism, further experiments were conducted in APP transgenic mice. It was found that Rd could also improve the learning and memory ability of APP transgenic mice by inhibiting inflammation [17]. At the same time, we also tried to prove that AD pathology of the olfactory bulb, spinal cord, and telencephalon besides hippocampus is sensitive to drug intervention.

## 2. Materials and Methods

### 2.1. Animals and Treatment

APP transgenic mice, 10 months old, provided by the Institute of Experimental Animals, Chinese Academy of Medical Sciences, were randomly divided into the APP group and the APP + Rd (10 mg/kg) group. The animals in the control group were wild-type mice of the same age and the same strain as APP transgenic mice. Rd administration regimen for the APP transgenic animal model: the experiment began with doses of Rd (10 mg/kg) treatment with intraperitoneal injection to animals once a day for six months.

### 2.2. Reagents

Mice anti-Tau-5 IgG was purchased from Millipore (Millipore, Bedford, MA, USA). Rabbit anti-Ser199/202 IgG and rabbit anti-Ser404 IgG were purchased from Biosource. Rabbit anti-Ser396 IgG was purchased from Abcam. Rabbit anti-GSK-3*β* IgG was purchased from Sigma. Rabbit anti-Ser9 IgG was purchased from Cell Signaling Biotechnology. Rabbit anti-Tyr216 IgG was purchased from Santa Cruz. Rabbit anti-P25 IgG and rabbit anti-P35 IgG were purchased from Cell Signaling Biotechnology.

### 2.3. Morris Water Maze

In the Morris water maze experiment, rats swam in the pool until they found a platform hidden underwater. Every morning and afternoon, the training lasted for 3 days. Hidden platform search data were obtained, reflecting the learning ability of rats. On the fourth day, the platform was removed. The number of times the rats crossed the target (the original platform position) within 60 seconds was measured as exploratory experimental data, reflecting the memory ability of the rats.

### 2.4. Western Blot Analysis

The frozen tissue was extracted from the spinal cord, olfactory bulb, and telencephalon, and the protein concentration was determined according to the instructions of the BCA kit (Kang Wei Biological Co., Ltd). Then, appropriate gel concentration was selected according to the molecular weight of the protein. Sampling with a microinjector, 1 x LB was added into the empty swimming lane. 1x running buffer was added, the voltage was set at 300 V, the current at 25 mA (a gel) or 36 mA (two gel), and the test was run to the bottom of the gel. 1x ^*∗*^TB is poured into the membrane transfer tank, and the membrane pad, filter paper, PAGE glue, PVDF membrane, filter paper, and transfer pad are placed from top to bottom. The voltage is 100 V, the current is 1250 mA, and the time depends on the molecular weight. The PVDF membrane with good membranes was immersed in 5% skimmed milk and closed, and the shaking table was shaken at room temperature for 1 hour. The first antibody was incubated at room temperature for 1 hour, then kept overnight in refrigerator at 4°C, and rewarmed at room temperature for 1 hour the next day. TBST is used to wash the membrane three times, 10 minutes each time. The second antibody is incubated, and 5% skimmed milk is used to allocate the second antibody, which was incubated in a shaking bed at room temperature for 1 hour. Then, TBST is used to wash the membrane twice, 10 minutes each time. 1x TBS is used to wash the membrane once, 10 minutes each time. The A and B solution of the chemiluminescence liquid is mixed in 1 : 1 ratio, dropped on the surface of the PVDF membrane which is incubated on the antibody, and blown evenly with the pipette, so that it can react on the membrane surface for about 1–3 minutes. Then, it was put into the gel imaging instrument to scan the film, the pictures was collected in the right time, and was preserved. Data were collected and analyzed statistically. This procedure was approved by the medical laboratory animal management committee.

### 2.5. Statistical Analysis

The final data were expressed as the mean ± SEM. SPSS 16.0 software was used for data analysis. Single factor analysis of variance was used for comparison between groups. Statistical significance of the results was evaluated at a level of *P* < 0.05.

## 3. Results

### 3.1. Rd Ameliorates Learning and Memory Ability in APP Transgenic Mice

Morris water maze was used to evaluate spatial memory. In the acquisition experiments, quantitative escape latencies indicated that after training, APP transgenic mice took longer to find the platform than did wild-type mice (*P* < 0.05), while this prolongation of latency was significantly shortened by Rd at the dose of 10 mg/kg (*P* < 0.05). In the probe trials, the number of crossing over a platform position for 60 s was used to estimate performance. The results showed that compared with the wild-type group, the time period that the APP transgenic mice crossed over the platform position for 60 s reduced significantly (*P* < 0.05). Compared with the APP transgenic group, the time period that mice in the Rd 10 mg/kg group crossed over the platform position for 60 s increased significantly (*P* < 0.05). Compared with the APP transgenic group, the time period of crossing over the platform position for 60 s in the APP transgenic mice + NS group had no obvious change.

### 3.2. Rd Reduced the Expression of Phosphorylated Tau Protein in the Olfactory Bulb, Spinal Cord, and Telencephalon of APP Transgenic Mice

**Figure 1:**
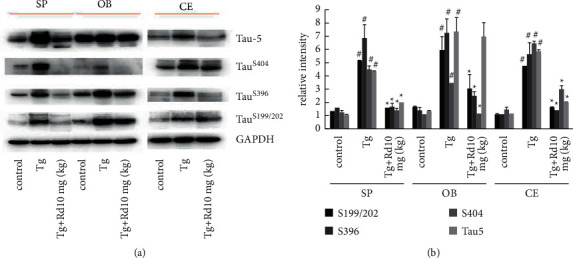
10 mg/kg Rd reduced the expression levels of phosphorylated tau protein in the spinal cord, olfactory bulb, and telencephalon in APP transgenic mice. (a) The expression of phosphorylated tau protein (S199/202, S396, S404, and Tau5) in the spinal cord, olfactory bulb, and telencephalon of APP transgenic mice was significantly higher than that in the control group. 10 mg/kg Rd can reduce the expression of phosphorylated tau protein (S199/202, S396, and S404) in the spinal cord, olfactory bulb, and telencephalon of APP transgenic mice. Furthermore, Rd could reduce the expression of phosphorylated tau protein (Tau5) in the spinal cord and telencephalon of APP transgenic mice. (b) Statistical histogram of Figure 1(a). ^*∗*^*P* < 0.05 vs. the Tg group, ^#^*P* < 0.05 vs. the control group.

Characterization of phosphorylated tau protein is a valuable way to assess the pathological changes in the olfactory bulb, spinal cord, and telencephalon. To determine the effect of Rd on the production of phosphorylated tau protein, the expression of phosphorylated tau proteins (S199/202, S396, S404, and Tau5) was detected by western blot using the specific antibody (Figure 1(a)). The expression of phosphorylated tau protein (S199/202, S396, S404, and Tau5) in the spinal cord, olfactory bulb, and telencephalon of APP transgenic mice was significantly higher than that in the control group. There was statistical significance in the group with *P* < 0.05 vs. the control group. 10 mg/kg Rd can reduce the expression of phosphorylated tau protein (S199/202, S396, S404) in the spinal cord, olfactory bulb, and telencephalon of APP transgenic mice, ^*∗*^*P* < 0.05 vs. the Tg group, with statistical significance. 10 mg/kg Rd could reduce the expression of phosphorylated tau protein (Tau5) in the spinal cord of APP transgenic mice and the Tau5 in the telencephalon, ^*∗*^*P* < 0.05 vs. the Tg group, which had statistical significance, but had no effect on the expression of phosphorylated tau protein (Tau5) in the olfactory bulb, and there was no statistical significance with *P* < 0.05. These results demonstrated that Rd could significantly decrease the deposition of phosphorylated tau protein ((S199/202, S396, and S404), Figure 1(b)).

### 3.3. Rd Reduced the Expression of Tyr216 in the Spinal Cord, Olfactory Bulb, and Telencephalon of APP Transgenic Mice

GSK-3*β* is a molecule of PI3K/Akt signaling pathway. Activated Akt combined with GSK-3*β* induces GSK-3*β* to transfer to the cell membrane, phosphorylates its active site N-terminal Ser9 and inactivates GSK-3*β*, thus affecting the downstream substrates such as tau protein. Several methods have been used to design and synthesize a series of derivatives, aiming at simultaneously regulating neuronal calcium channels and GSK-3*β* to produce effective targets for the treatment of Alzheimer's disease [21].

**Figure 2:**
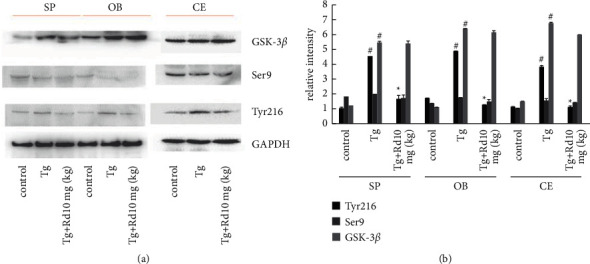
10 mg/kg Rd reduced the protein expression levels of Tyr216, Ser9, and GSK-3*β* in the spinal cord, olfactory bulb, and telencephalon of APP transgenic mice. (a) The expression of Tyr216 in the spinal cord, olfactory bulb, and telencephalon of APP transgenic mice was significantly higher than that of the control group. 10 mg/kg Rd could decrease the expression of Tyr216 in the spinal cord, olfactory bulb, and telencephalon of APP transgenic mice, which was statistically significant. Rd had no effect on the expression of Ser9 and GSK-3*β* in the spinal cord, olfactory bulb, and telencephalon, but there was no significant difference between the two groups (*P* > 0.05 vs. the Tg group). (b) Statistical histogram of Figure 2(a). ^*∗*^*P* < 0.05 vs. the Tg group, ^#^*P* < 0.05 vs. the control group.

To further elucidate the mechanism of Rd in inhibiting tau hyperphosphorylation in the spinal cord, olfactory bulb, and telencephalon of APP transgenic mice, we assessed the expressions of GSK-3*β* and pGSK-3*β* (Figure 2(a)) by western blot. The expression of Tyr216 in the spinal cord, olfactory bulb, and telencephalon of APP transgenic mice was significantly higher than that of control group, ^#^*P* < 0.05 vs. the control group. 10 mg/kg Rd could decrease the expression of Tyr216 in the spinal cord, olfactory bulb, and telencephalon of APP transgenic mice, which was statistically significant. ^*∗*^*P* < 0.05 vs. the Tg group. 10 mg/kg Rd had no effect on the expression of Ser9 and GSK-3*β* in the spinal cord, olfactory bulb, and telencephalon, but there was no significant difference between the two groups (*P* < 0.05 vs. the Tg group, Figure 2(b)).

### 3.4. Rd Reduced the Expression of P25 and Increased the Expression of P35 in the Spinal Cord, Olfactory Bulb, and Telencephalon of APP Transgenic Mice

Increased P25, a proteolytic fragment of the regulatory subunit P35, is known to induce aberrant activity of cyclin-dependent kinase 5 (CDK5), which is associated with neurodegenerative disorders, including AD. Therefore, inhibition of the CDK5 pathway may represent a novel therapeutic strategy against A*β*-induced neurodegeneration [22, 23].

**Figure 3:**
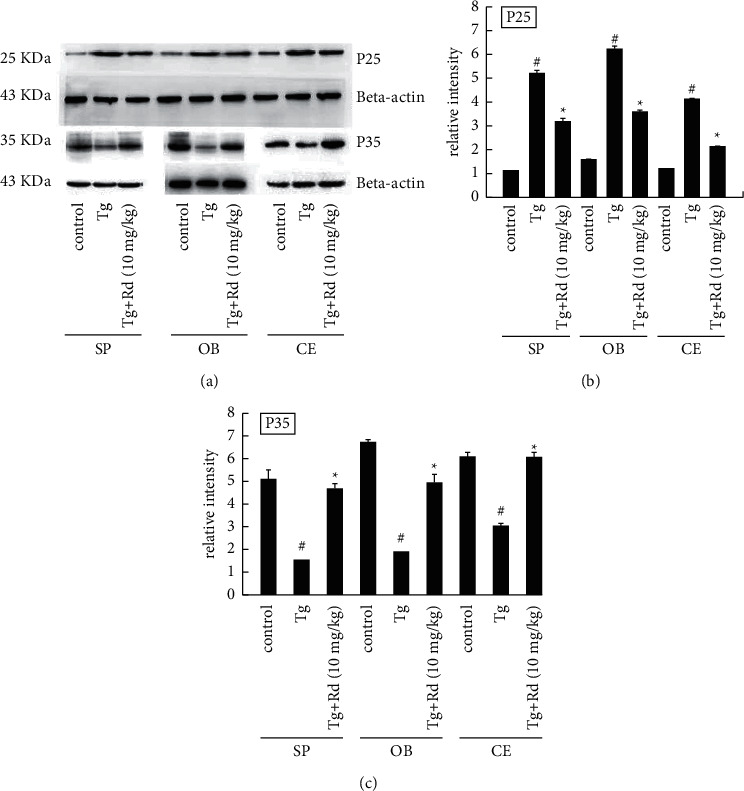
Effect of Rd on the protein expression levels of CDK5/P25 and CDK5/P35 in the olfactory bulb, spinal cord, and telencephalon of APP transgenic mice. (a) The expression of CDK5/P35 in the spinal cord, olfactory bulb, and telencephalon of APP transgenic mice was significantly less than that of the control group. 10 mg/kg Rd could increase the expression of P35 in the spinal cord, olfactory bulb, and telencephalon of APP transgenic mice, which was statistically significant. The expression of CDK5/P25 in the spinal cord, olfactory bulb, and telencephalon of APP transgenic mice was significantly higher than that of the control group. 10 mg/kg Rd could decrease the expression of CDK5/P25 in the spinal cord, olfactory bulb, and telencephalon of APP transgenic mice, which was statistically significant. (b) Statistical histogram of P25. (c) Statistical histogram of P35. ^*∗*^*P* < 0.05 vs the Tg group, ^#^*P* < 0.05 vs. the control group.

To further elucidate the mechanism of Rd in inhibiting tau hyperphosphorylation in the spinal cord, olfactory bulb, and telencephalon of APP transgenic mice, we assessed the expressions of CDK5*/P25/P35* (Figure 3(a)) by western blot. The expression of *P25* in the spinal cord, olfactory bulb, and telencephalon of APP transgenic mice was significantly higher than that of the control group, ^#^*P* < 0.05 vs. the control group. 10 mg/kg Rd could decrease the expression of *P25* in the spinal cord, olfactory bulb, and telencephalon of APP transgenic mice, which was statistically significant. ^*∗*^*P* < 0.05 vs. the Tg group. The result of P35 was the opposite. The expression of P35 was significantly less than that of the control group, ^#^*P* < 0.05 vs. the control group. 10 mg/kg Rd could increase the expression of P35, which was statistically significant. ^*∗*^*P* < 0.05 vs. the Tg group (Figures 3(b) and 3(c)).

## 4. Discussion

It is well known that the pathological manifestation of AD is the entanglement of A*β* deposition and phosphorylated tau. At present, the recognized pathological manifestation of AD occurs in the hippocampus and cortex. However, no detailed description of amyloid beta plaques and phosphorylated tau protein loads in the spinal cord, olfactory bulb, and telencephalon has been provided so far. With the development of research, olfactory memory disorders were also found in 3×Tg-AD mice [24]. Studies have shown that olfactory impairment is a major part of cognitive impairment in AD, and olfactory impairment is highly correlated with A*β* deposition and tau hyperphosphorylation [7], especially OB [25], which occurs outside the cortex and hippocampus. OB is the first site of brain olfactory information processing, and its disorder is related to AD. In addition, neuronal dysfunction, including all regions and neuron types in OB, is an early event of AD [26]. Moreover beta-amyloid plaques can also be seen in the human spinal cord, most notably in familial AD cases [2]. As an integral part of the central nervous system, the spinal cord has the same molecular mechanism for the formation of beta-amyloid plaques, as shown in various transgenic mouse models, which overexpress APP or APP mutations and PS1 mutations [11, 27]. In this study, we characterized spinal cord, olfactory bulb, and telencephalon pathology in one of the commonly used transgenic mouse models, which expresses mutated APP. First, we observed the expression of phosphorylated tau protein by western blot. Characterization of phosphorylated tau protein is a valuable way to assess the pathological changes in the spinal cord, olfactory bulb, and telencephalon. The expression of phosphorylated tau protein (S199/202, S396, S404, and Tau5) in the spinal cord, olfactory bulb, and telencephalon of APP transgenic mice was significantly higher than that in the control group. Of note, phosphorylated tau protein deposits in the spinal cord, olfactory bulb, and telencephalon have also been reported in the human AD brain [3, 10].

The potential cause of olfactory dysfunction may be the deposition and accumulation of A*β* and tau proteins in the OB, spinal cord, and telencephalon [28]. Studies have shown that A*β* deposition occurs in the OB, spinal cord, and telencephalon of 3–4 months old, and is associated with olfactory and cognitive deficits in AD mice [29]. Phosphorylated tau protein plays an important role in the neurotoxic pathology of AD. In AD patients and transgenic mice, the accumulation of phosphorylated tau protein and soluble A*β* in the OB was also strongly correlated with very early olfactory dysfunction [30]. On the contrary, the decrease of phosphorylated tau protein and A*β* improved olfactory dysfunction observed in AD transgenic mice [29]. Therefore, Rd pretreatment reduced the expression of phosphorylated tau protein in the OB, spinal cord, and telencephalon of APP transgenic AD mice, as confirmed by current results. It has been found that the olfactory system of AD patients showed more NFT than plaque [24]. Therefore, in this study, we examined the expression of phosphorylated tau protein in the OB, spinal cord, and telencephalon. In addition, Rd pretreatment significantly reduced tau and tau phosphorylation levels at S199/202, S396, and S404 sites in the OB, spinal cord, and telencephalon.

It is well known that GSK-3*β* is a proline-directed serine/threonine kinase with structural activity, which enhances tau phosphorylation and regulates the amyloidosis of APP by regulating the expression of BACE and the function of gamma-secretase *in vivo* and *in vitro* [31]. In addition, our previous studies have confirmed that Rd reduces tau phosphorylation by inhibiting GSK-3*β* activity in the cortex and hippocampus of AD rats [32]. In the OB, spinal cord, and telencephalon, Rd also decreased the activity of pGSK-3*β* and Try216.

In addition, as the most important dephosphatase, CDK5 is the key upstream kinase in the pathological pathways of A*β* and tau. Therefore, we continue to explore the expression of cyclin-dependent kinase 5 (CDK5). CDK5, a cyclic-dependent protein kinase, has been found to be closely related to the occurrence of Alzheimer's disease. CDK5, a protein kinase, can acidify many subunits, which are indispensable to the function of mature neurons, such as the maturation and differentiation of neurons, synaptic function, memory consolidation, glial cell regeneration, and cerebellar function. CDK5 is different from other members of the cyclic-dependent protein kinase family. It is mainly active in the neurons of the late mitotic stage because its activators P35 and P39 are neuron-specific and only locally expressed in the nervous system. Because of the increase of intracellular calcium concentration, P35 can be hydrolyzed to P25 by calpain, which can form a more stable and harmful complex with CDK5-CDK5/P25. This complex can lead to high phosphorylation of many subunits, which can lead to a variety of pathological changes in Alzheimer's disease, such as the formation of senile plaques and neurofibrillary tangles and synaptic dysfunction. In view of the role of CDK5 in the pathogenesis of Alzheimer's disease, it is possible to treat Alzheimer's disease by interfering with the high activity of CDK5. It was found that CDK5 inhibitors and calpain inhibitors have certain effects, but they have their own limitations. Therefore, in this study, we examined the expression of P25/P35 in the OB, spinal cord, and telencephalon. In addition, Rd pretreatment significantly reduced the expression of P25 and increased the expression of P35 in the OB, spinal cord, and telencephalon.

In conclusion, our findings provide the first evidence that ginsenoside Rd is capable of inhibiting A*β*-induced tau phosphorylation by altering the functional balance of GSK-3*β* and CDK5/P25 in the OB, spinal cord, and telencephalon. Considering that only ginsenoside Rd pretreatment is effective for suppression of tau phosphorylation, ginsenoside Rd may be used as a promising drug for preventing the progression of early AD, which needs further basic experiments and clinical trials to clarify.

## Data Availability

The data used to support the findings of this study are available after two years after publication.

## Abbreviations

AD: Alzheimer's disease
Rd: Ginsenoside Rd
GSK-3*β*: glycogen synthase kinase-3*β*
CDK5: Cyclin-dependent kinase 5.
